# The electromagnetic bio-field: clinical experiments and interferences

**Published:** 2012-06-18

**Authors:** G Burnei, D Hodorogea, I Georgescu, Ş Gavriliu, I Drăghici, D Dan, C Vlad, L Drăghici

**Affiliations:** * “M.S. Curie” Emergency Pediatric Hospital, Bucharest, Romania; ** Surgery Department of „Sf. Ioan” Clinical Emergency Hospital, Bucharest, Romania

**Keywords:** bio-structures, magnetic field, living organisms, animals

## Abstract

**Introduction:** One of the most important factors is the technical and scientifically rapid development that is continually modifying the world we live in and polluting it with electromagnetic radiations. A functional and structural influence of magnetic and electromagnetic field on living organisms is presented in the literature by many performed experiments.

**Material and methods:** The notion of bio-field represents the electromagnetic field generated by the bio-structures, not only in their normal physiological activities but also in their pathological states. There is a tight interdependency between the bio-field and the bio-structure, which respects the primary notion of an electromagnetic field given by the Maxwell-Faraday laws, in which, the electromagnetic phenomena are simplified to the field variations. These variations can be expressed in a coherent differential equation system that bounds the field vectors to different space points at different time moments.

**Results:** The living organisms cannot contain electrostatic and magneto-static fields due to the intense activity of the bio-structures. The biochemical reactions that have high rhythms and speeds always impose the electrodynamics character of the biologic field that also corresponds to the stability of the protein molecule that can be explained only through a dynamic way.

The existent energy is not considered an exciting agent, and it does not lead to any effects.

**Conclusions:** The parameters of these elementary bio-fields cannot yet be fully known due to technical reasons. The biological structures are very complex ones and undergo continuous dynamical activity. That is why the calculus model should be related to the constant dynamics, nowadays being very difficult to express.

## Introduction

The study of the influence exercised by the manmade electromagnetic fields began to concern the scientists and especially the biologists worldwide, mainly in the last two decades. 

One of the most important factors is the technical and scientifically rapid development that is continually modifying the world we live in and polluting it with electromagnetic radiations. 

This is why, the main reason for the study of its effects, has become actual than ever and preoccupies the scientists from the point of view of the consequences that we will have to endure [**1**]. Moreover, the impressive technological development at the end of the 20th century and in the beginning of the 21st century deeply influenced all the medical domains, by introducing the electromagnetic waves techniques into the day-by-day practice. All these techniques have significantly marked many diagnostic procedures [**2**]. 

Nowadays, the high performance obstetrical ultrasonography allows us to detect the congenital diaphragmatic defect ever since the 25th week of gestation. Therefore, the echocardiography and amniocentesis became two rather complementary methods of obstetrical diagnostic that are meant to exclude the chromosomal anomalies and cardiac malformations [**3**].

There is no doubt that this kind of energy becomes a continually increasing ecological factor that needs to be studied in time, due to its important influence on every living organism.

Scientists and especially the Physicists formulated hypotheses regarding the way in which the external electromagnetic fields interact with the living organisms. They have mainly highlighted the possibility of inducing several physics phenomena at an atomic level such as: magnetic moment orientation, atomic and molecule oscillation reduction, etc. They do not refer to the fact that there are macromolecules of great specificity in an organism, integrated in very complex structures. Due to these facts, the electromagnetic fields will not only produce primary effects in the living organisms but also many other secondary effects. The primary effects are not enough to explain the diversity of the organisms’ responses to the interaction with the electromagnetic fields. 

## Objectives

Starting from the Maxwell’s Electromagnetic Field Theory, that opened vast horizons on the interpretation and research, not only in Physics but also in Biology, a new theory was elaborated, regarding the interaction of the electromagnetic field with the living organisms.

It is a known fact that a moving electron produces an electromagnetic field and that the metabolism is a precisely chained oxide reduction process in which the electrons carried by the specific respiratory enzymes generate oxide reduction electric potentials, therefore electromagnetic fields that generate the biochemical process. These fields generated by the living organisms were named “bio-fields”.

The electromagnetic bio-field notion (EMF) became a necessity due to the need of interpreting the experimental laboratory results, on the effects that small intensity electromagnetic fields have, when used on treating living organisms.

The need for this notion was also for clarifying the different biological phenomena that take place at a cellular and sub cellular level and that are not yet known.

## Material and method

The notion of bio-field represents the electromagnetic field generated by the biostructures not only in their normal physiological activities but also in their pathological states. The generating element is the protein macromolecule. Even if this macromolecule is the main generator of the field, there are also some other elements that contribute to it, such as ions and electric charges that belong to the bio-structures.

The structure of the magnetic bio-field does not vary much from that of the non-biological electromagnetic field. It is an electromagnetic field with two perpendicular components and both perpendicular on the propagation direction. There is a tight interdependency between the bio-field and the bio-structure because a certain bio-structure creates a certain bio-field and a certain bio-field influences a bio-structure in a specific way.

Unlike the natural electromagnetic fields or manmade fields, the bio-field is highly distorted because it is generated by highly asymmetric structures. These structures also have very important and permanent functional variations.

This interdependency respects the primary notion of an electromagnetic field given by the Maxwell-Faraday laws, in which the electromagnetic phenomena are simplified to the field variations. These variations can be expressed in a coherent differential equation system that bounds the field vectors to different space points at different time moments.

## Results

Any variation of the field in a point P directly influences the points from its vicinity. The perturbation is transmitted from one point to another in finite time intervals. The electromagnetic field propagates in a wavelike form, transporting a certain quantity of energy. This electromagnetic energy can also transform into mechanical, chemical or thermal energy. The speed of this perturbation was calculated by Maxwell and it is the speed of light in vacuum: 3,10¹⁰cm x sˉ¹. 

In the case of the radiation, the electromagnetic energy is independent of the charge density (φ) and of currents (J→). 

The electromagnetic field emits a certain amount of energy in space and therefore acts with a force on the charges that are in the field. This force is named “Lorenz force” and has the following formula: 

F= qE→ + qV→ x B→

Lorenz force has the following components:

1) electric components qE→ ;

2) magnetic components qV→ + B→, considered as a consequence of the electric components. 

It can be observed that the first part of the equation represents the force with which the electromagnetic field, and therefore the bio-field, acts upon an electric charge. This force is independent of the speed of light and has the same direction as E→.

The second part of the equation, which is the relativist result of E→, is perpendicular on the charge direction and on the direction of B→.

Conventionally, when E→ ≠ 0 and B→ = 0, we consider the electric field and when E→ = 0 and B→ ≠ 0, we consider the magnetic field.

The electromagnetic bio-field laws are contained in Maxwell’s equations that completely describe the dynamic aspect of the field. If the terms that contain the differentials with respect to time are constant, they become zero (the derivative of a constant is zero).

These are the conventional conditions in which we consider the electrostatic and magneto static fields.

The living organisms cannot contain electrostatic and magneto static fields due to the intense activity of the biostructures. The biochemical reactions that have high rhythms and speeds always impose the electrodynamics character of the biologic field that also corresponds to the stability of the protein molecule that can be explained only through a dynamic way.

## Discussions

The terms electrostatic and magneto static are conventional. They make the calculations easier but they will not be important in Biology, where the dynamic element is so relevant. Different professional societies continue to recommend the conventional methods based on the X-rays, ultrasounds or nuclear magnetic resonance [**4,5**]. The penetration capacity of the electromagnetic waves is used in imagistic investigation methods used for both humans and animals: the X-rays diagnose the bowl obstructions and the ultrasonography confirms the presence of the tumors [**6**].

### Bio-field and electromagnetic radiation

One can say that the electromagnetic radiation travels through the space. The amortization of these waves, produced by oscillators, can be absorbed by regions containing electric charges or other fields that can annihilate them through destructive interference. There is also the possibility of amplifying them through constructive interference. These interferences depend on the amplitude of the oscillations. 

Due to the distribution of the oscillating dipole moments and magnetic moments, the protein molecule has its own means of decreasing or amplifying the radiation. Moreover, the field parameters variation can turn the field to a homogenous one, but is unable to make this homogeneity perfect.

Considering that the protein macromolecule bio-field reflects very well its structure, it represents the way of transferring the energy from one bio-structure element to another. This transfer is made by using highly encoded radiation. This encoding has a very important role in the processes that takes place at the level of the cells and of the protein macromolecule. The highly informational content of the protein macromolecule and the direct result of its structure is the base for the immense variability of the functional actions, such as the biochemical chain reactions that are automated and flawless. 

The nervous impulse triggers these processes; the protein macromolecule structure with its highly informational content ensures that the written program is well executed.

The problem of the bio-field generated by the biologic structures can raise the problem of their stability; this very important problem being related to that of the absorption and emission of the radiation by the protein molecule. Moreover, one more matter is very important, ensuring a biological reactivity. This opposes to a high stability, which would diminish this reactivity. 

Both issues, the necessary stability to assure the function’s specificity, as long as a certain degree of instability exists, are related to the biological reactivity and the way the energy is transferred. 

The energy transfer is closely related to the bio field; through this, the macromolecule will receive and cede energy; of course, ceding and receiving energy by the protein macromolecule will be accomplished in quantum conditions, so that the macromolecule will keep its stability energy and in the same time will be able to get the energy for its activity.

It is well known that the bio structures are asymmetric, if taking into account their fundamental chemical components up to the more complex structure of the protein macromolecule. This asymmetry is not random at all, but it has an important biological significance [**7**].

It is known that in all the organic matter the carbon atom is the basic element, which the bio structures rely on. The carbon atom is tetravalent and these valences have the most uniform space distribution: they form a tetrahedron having the carbon atom in the middle.

In case four identical radicals occupy the valences, the tetrahedron is regulated, for example: CH4, CCl4.

The carbon atom also has the tendency to saturate the valences with four different radicals. In this case, the tetrahedron that forms is completely irregular and so the carbon atom is asymmetric.

The amino acids that are of great importance in biochemistry and form the big protein molecule by polymerization are also in the category of the chemical compounds with asymmetric carbon. This is the reason for the biologic asymmetry.

Besides this, the carbon atoms tend to form covalent chains, and the protein forming is possible through amino acid chains, fibers and helixes. There are proteins that have 200 to 300 amino acids or even more. Due to the asymmetries of the carbon atom, amino acids have an optical activity that depends on the positioning of the amino group with respect to the carbon atom.

Being a high polymer of the amino acids, the protein molecule, each of them being asymmetric, will also have a high asymmetry. 

The biological molecule asymmetry is easier to understand by introducing the terms of “electric symmetry and asymmetry” and “magnetic symmetry and asymmetry”.

It is known that each molecule has a dipole moment that is calculated with the following equation:

P = e x l

in which:

p = dipole moment

e = electric charge (+ e and - e)

l = distance between the electric charges

If l is smaller than R₁ and R₂, the electric charges ensemble is called dipole (double pole). 

A chemical substance whose molecule atoms are symmetrically stirred has zero dipolmoment, for example: CO₂ or CH₄. Having asymmetric carbon and opposite electrical charges in different centers of the molecule, the amino acids have high electrical dipole moments and through this, they enhance the permittivity of the water in which they are dissolved.

It has been established through thermodynamic calculus, that there can only be transitory neutral molecules and in small quantities, in the solution of aminoacids. 

At the same time, the dissolution of aminoacids in water causes the appearance of intense forces in the solvent’s molecule (ions-dipole forces). For example, the dissolvance of a molecule of glycol (75 g with volume of 47 cm³) can cause the decrease of the solution’s volume to 43 cm³ (a phenomenon called electrostriction). 

The asymmetric molecules are generally polarized; it is assumed that the center of gravity of the „+” charges does not meet the one for the „−” charges.

The polarization of the asymmetric molecule depends on T° (Kelvin).

Apart from the electrical asymmetry, there can also be a magnetic asymmetry. If the electrical symmetry depends on the distribution of charges within the molecule, the magnetic moment depends on the distribution of the J→ currents, which define the magnetic moment M.

It is considered that each moving charge determines a current that is dependent on S, then the current has the magnetic moment M = J→ x S (within the atom, the moment is provided by the current caused by the electrons).

In atoms and molecules, the overall magnetic moment depends on how the orbital and spin magnetic moment is compensated. When it is compensated, the molecules or atoms do not have a magnetic moment (equals zero); when the molecule has an even number of electrons the moment is zero, but the rule has exceptions [**8**].

The asymmetry is important because it is one of the forces of electrical and magnetic asymmetry, which represents both a condition and a method of the molecule’s reactivity. 

Generally, the substances introduced in the electrical field can suffer two opposite processes: a) conductivity, b) polarity.

In the case of conductivity, if the substance has free charges (eˉ, p⁺, ions), they will start moving. 

In the case of polarity, the charges will move towards a position of equilibrium, with the molecule being oriented in a specific way or even performing small movements. 

If a molecule or an atom with spherical symmetry is introduced in the electrical circuit, the electronic cloud shifts from the nucleus and produces the asymmetry. The possibility for energy transfer depends on the variability of the electrical and magnetic molecules.

Through their extreme functional mobility, the protein molecules generate electromagnetic micro-fields just as variable and numerous as the generating proteins.

The structural asymmetry of the protein molecules ensures a certain level of electrical polarity, because the center of gravity of the positive charges in the molecules does not meet the one of the negative charges. However, at the same time and apart from the electrical asymmetry there is also a magnetic asymmetry, depending on the distribution of currents defining the magnetic moment. Therefore, it is considered that this electromagnetic asymmetry ensures a certain level of molecule reactivity and particularly of energy transfer. 

It is impossible to deny the fact that these bio-fields interact and play a fundamental part in the energy transfer from one structural element to another, from one molecule to another, from one organ to another. It is also likely that a communication of codified information is realized this way, essential for the complex functional automatism of the animal organism. Hence, these biological micro-fields can represent factors of functional connection between the component parts of the organism, but, at the same time, can be actively radiated by the living structures and become a method of connection between individuals.

Contemporary Physics deals with the field as a vectorial property of space through which an action vector can be attached to each belonging point. If the vectorial representation of the field is admitted, it is highly implied that this field represents o sum of actions, an energy or rather a working capacity in different forms.

In addition, at this moment, in Physics, *„the action quantum”* is regarded as a particle of the field and it is considered responsible for the interactions taking place within the field or between the crossing fields. These particles *highlight* the electromagnetic field, either biologically or magnetically generated by the inert substances [**9**]. 

The relation between mass and energy proves that the production of energy is related, indefinitely, to the existence of material mass and the presence of a mass undoubtedly implies the possibility of energy release through a certain method. 

The electromagnetic field, which, as mentioned before, represents energy, also possesses, as proven, the qualities of matter: mass, impulse, inertia, etc.

Moving on to the living structures, the way they produce energies can be easily observed but, in this case, there are two specific factors for living matter, which are:

a) the released energy depends on the structure of the respective tissue and it is produced at a slower pace, but in accordance to the known laws of conservation;

b) the energy is released by these structures in specific parts of the bio-chemical enzyme chain, composed of a number of stages, specific to each Physico-chemical process. 

Perhaps it would be useful to embrace the notion „energy of structure” for the first case, given the fact that the energy released varies according to the type of the respective structure and, for the second case, the notion „energy of structure” because the size and distribution of the released energy depends of a certain structure.

Therefore, the living molecule possesses, depending on its structure, a bio-field and sometimes a bio-radiation, hence the energy and the generated bio-field of biostructures will also have an influence on the biostructures.

It has been considered until now that the bio-field is generated by biostructures, by the specific activity of the living substance and has been alleged that it is the product of charges configuration and their movement during the biological activity.

Subsequently, we will try to complete this hypothesis with considerations particularly related to the helical molecules of the proteins, which are also known to be composed of a well-established alignment of amino acids, having radicals connected through peptide links.

Other connections can also be formed along these catenaries, or in-between the peptide chains, for instance disulphide, through which the electronic excess of the nitrogen atom is compensated. The hydrogen link from the peptide molecule is stronger than that of the hydrogen bridge in the water molecule. There are also ionic links between the carboxylic and amine connections, among which there is a force of attraction and some apolar links, where the van der Waals force is applicable.

Hence, the amino acids containing in their molecule opposite electrical charges have high electrical dipolmoments, through which they enhance the permittivity of the water. As the protein molecule is composed of an alignment of amino acids, it is considered that the distribution of their electrical dipolmoments forms a network around the protein molecules, whose variation is responsible for the creation of a bio-field. 

The energy transfer in a molecular system is not possible if the dipolmoments have very slight variations or none. This hypothesis brings together both the stability requirement and the reactivity, requested by the protein molecule, necessary for the energy exchange within the existing system. While examining the previous statements, it is essential to make the following observation: the biological energy is released gradually, in specific parts, on the stairs of the enzyme chain, no matter which one [**10**].

While reflecting on this method of energy release, it is almost impossible not to compare it with the energy quantification method, introduced for the first time by Plank and used nowadays in Physics. Inherently, the question whether this is a bio quantum or not arises, an indivisible amount of energy, united, that would constitute a necessary minimum in order to obtain activity energy and thus trigger the enzyme chain reaction.

The same reactions take place among the bio-fields in which the photon (quantum) plays an important part in action and interaction. Therefore, bioenergetics, admitting this new notion of bio quantum, might proceed to studying energies at a molecular level, meaning to biological energy, which could be evaluated through integrals of bio quantum. Related to these, it must not be forgotten that proteins possess an informational baggage encrypted in an engrams transmitted from the far ecological ages, an experience misted by the depth of times. Given this engrams, the living organisms have a high energetic efficiency. Rigorous studies performed on inferior organisms indicated an efficiency of 85 % - 90%, which represents a proof of the improvement of living structures with regard to the use and distribution of energy.

In a future study, we would like to analyze the LASER techniques and applications in the no suture surgery [**11**]. Lobe and Schropp have used KTP/532 Laserscope at approx. 12-15 W or Nd:YAG at approx. 40-60 W for obtaining a hepatic excision with no suture in haemostatic conditions [**12**].

## Conclusions

The parameters of these elementary bio-fields cannot be fully known yet due to technical reasons. The biological structures with high organization undergo continuous activity and therefore the calculus model should be considered through its dynamics, which might appear extremely difficult. If the parameters of the bio-fields cannot still be calculated, the resultant of the bio-fields’ cells forming an organ, can be calculated at least with approximation and, by using proper tools, can be experimentally verified.
